# Genome-Wide Association Study Identifies Multiple Susceptibility Loci for Malignant Neoplasms of the Brain in Taiwan

**DOI:** 10.3390/jpm12071161

**Published:** 2022-07-18

**Authors:** Jang-Chun Lin, Yi-Chieh Wu, Fu-Chi Yang, Jo-Ting Tsai, David YC Huang, Wei-Hsiu Liu

**Affiliations:** 1 Department of Radiation Oncology, Shuang Ho Hospital, Taipei Medical University, Taipei 11031, Taiwan; 13451@s.tmu.edu.tw (J.-C.L.); 10576@s.tmu.edu.tw (J.-T.T.); 2 Department of Radiology, School of Medicine, College of Medicine, Taipei Medical University, Taipei 11031, Taiwan; 3 Department of Neurological Surgery, Tri-Service General Hospital, National Defense Medical Center, No.325, Section 2, Cheng-Kung Road, Taipei 11490, Taiwan; stundinterne@gmail.com; 4 Department of Neurology, Tri-Service General Hospital, National Defense Medical Center, No. 325, Section 2, Cheng-Kung Road, Neihu 114, Taipei 11490, Taiwan; fuji-yang@yahoo.com.tw; 5 Department of Medical Physics, Duke University, Durham, NC 27708, USA; yh126@duke.edu; 6 Department of Surgery, School of Medicine, National Defense Medical Center, Taipei 11490, Taiwan

**Keywords:** brain cancer, glioma, SNP, LMF1, RBFOX1

## Abstract

Primary brain malignancy is a rare tumor with a global incidence of less than 10 per 100,000 people. Hence, there is limited power for identifying risk loci in individual studies, especially for Han Chinese. We performed a genome-wide association study (GWAS) in Taiwan, including 195 cases and 195 controls. We identified five new genes for malignant neoplasms of the brain: EDARADD (rs645507, 1p31.3, *p* = 7.71 × 10^−5^, odds ratio (OR) = 1.893), RBFOX1 (rs8044700, *p* = 2.35 × 10^−5^, OR = 2.36), LMF1 (rs3751667, *p* = 7.24 × 10^−7^, OR = 2.17), DPP6 (rs67433368, *p* = 8.32 × 10^−5^, OR = 3.94), and NDUFB9 (rs7827791, *p* = 9.73 × 10^−6^, OR = 4.42). These data support that genetic susceptibility toward GBM or non-GBM tumors is highly distinct, likely reflecting different etiologies. Combined with signaling analysis, we found that RNA modification may be related to major risk factors in primary malignant neoplasms of the brain.

## 1. Introduction

Several different kinds of neoplasms occur in the brain and central nervous system (CNS), including cranial nerves and the spinal cord. Some brain neoplasms are benign lesions, whereas others are malignant, and whether a neoplasm is malignant depends on the doubling time during tumor cell proliferation. In other words, if a brain neoplasm is composed of slowly proliferating cells, it is a usually benign tumor. Conversely, if tumor cells grow and spread quickly, even in the midst of normal brain tissue, a brain malignancy is present. Regardless of benign or malignant status, brain tumors have similar effects on cranial neuropathy and brain injury, and patients experience dizziness, headache, seizure, and even paralysis. Moreover, patients with benign and malignant brain tumors have different prognoses. Epidemiological studies have revealed risk factors for primary brain tumors, including benign and malignant neoplasms. However, not much information is known about the epidemiology of brain neoplasms, even after a series of case–control analyses [1].

The International Classification of Diseases (ICD) was implemented more than 35 years ago. It has been an indispensable template for analysis of the epidemiology of brain neoplasms in the Taiwan Cancer Registry and worldwide. According to Cancer Registry Annual Report 2018 Taiwan, cases of primary malignant brain tumors accounted for 0.62% of all cancers, and the number of deaths from malignant brain neoplasms accounted for 1.25% of all deaths from malignancy. The most common histopathology of brain cancer is malignant glioma. Glioma is classified according to the World Health Organization (WHO) grading system as anaplastic astrocytoma (AA) or anaplastic oligoastrocytoma (AO) WHO grade III and glioblastoma (GBM) WHO grade IV [2]. In 2004 [3], a study in the United States of America established an incidence-trending model of adult glioma for different grades of gliomas, and this model showed a significant interaction between patient age and sex in GBM. Furthermore, sex correlates significantly with the year of diagnosis in AA, and with the year of diagnosis. There is also incident correlation between sex and race in OA.

In 2016, the WHO classification included genomic analysis as a novel approach for newly diagnosed glioma patients. Therefore, an increasing number of studies [4,5,6] have attempted to identify genetic risk factors for brain cancer through genome-wide association study (GWAS) approaches in which single-nucleotide polymorphisms (SNPs) have been found to have associations with brain malignancy, especially in malignant glioma [7,8]. An SNP is a variation at a single base pair among individuals, which may indicate a risk locus. Polygenic susceptibility of a glioma through GWAS supports the finding that SNPs at the following loci are risk factors for glioma: 17p13.1 (TP53), 11q23.3 (near PHLDB1), 8q24.21 (near CCDC26), 7p11.2 (near EGFR), 5p15.33 (near TERT), and 3q26.2 (near TERC) [8]. These SNPs suggest association with several molecular subgroups of glioma. SNPs from the 1000 Genomes Project combined with UK, German, and French GWASs have been confirmed as conferring susceptibility to glioma in populations of European ancestry. However, there is no credible, major project in the Han Chinese population assessing SNPs between benign and malignant brain neoplasms.

Therefore, we aimed to identify susceptibility loci to further evaluate benign and malignant brain neoplasm subgroups. We performed GWAS with the Taiwan Precision Medicine Initiative (TPMI) using study group data for patients with brain cancer (ICD-10: C70, C71) and those with benign neoplasms (ICD-10: D33) as controls of the Han Chinese population residing in Taiwan. The bulk of this article concentrates on SNPs associated with malignant neoplasms.

## 2. Materials and Methods

### 2.1. Genotyping and SNP Selection

The GWAS study comprised 390 patients with glioma ascertained through the TPMI and was approved by the Institutional Review Board of Tri-Service General Hospital with TSGHIRB No. 2-108-05-038. The protocol of the GWAS study was conducted according to the guidelines of the Declaration of Helsinki. Our research collected DNA for genotyping from peripheral blood. SNP detection utilized the Affymetrix Axiom Genome-Wide TWB 2.0 array plate, which was specially designed to identify disease-related SNPs or drug metabolism-related SNPs that represent the Taiwanese genotypic background. Genomic DNA was extracted from blood with the QIAamp DSP DNA Mini Kit in the QIA symphony platform (Qiagen, Hilden, Germany), and an assessment of this extraction process was performed with a NanoDrop One spectrophotometer (Thermo Fisher Scientific, Waltham, MA, USA). Next, signal file raw data were transformed into genotyping profiles using Analysis Power Tools. Quantile normalization was performed before GWAS analysis. The quality of genotyping was evaluated using PLINK. Unqualified SNPs were excluded, as they could not achieve either a genotype calling rate of >97% or a Hardy–Weinberg equilibrium of *p* > 0.00001. To exclude missing SNPs in a large proportion of the subjects, we filtered out SNPs with very high levels of missingness (>10%). SNPs that met the minor allele frequency threshold (>5%) were included. According to the common practice in the field of GWAS, a *p*-value < 5 × 10^−8^ was considered replicated, and a *p*-value < 10^−5^ was deemed to be suggestively significant. We adopted this value for the selection of brain neoplasm-related SNPs in our study.

### 2.2. Genotype-Phenotype Association Analysis

To evaluate the relationship between selected SNP (genotype) and brain neoplasm and its indices (phenotype), statistical significance tests, including the Pearson’s chi-square test and logistic regression analysis, were conducted. We determined eight SNPs that revealed a suggestively significant relationship with an ≥1 index of brain neoplasm at this step and further constructed a brain neoplasm-related SNP genotype score described in the following section.

### 2.3. Establishment of Brain Neoplasm-Related SNP Genotype Scores

After selecting a set of SNPs that revealed suggestively significant associations with brain neoplasm in our study population, we wondered whether having multiple SNPs generated an aggregating effect on brain neoplasm. We hypothesized that having a higher number of brain neoplasm-related SNPs denoted a higher risk of brain neoplasm. Therefore, we established a genotype score that added up the number of SNPs that carried unfavorable alleles in each subject. A similar scoring method has been used previously to predict cardiovascular disease risk in a large study of >5000 subjects [9]. Such a scoring model aims at examining the combined contribution of multiple SNPs toward a single disease. Each SNP produces two scores: zero for no unfavorable alleles (the homozygous genotype of the major allele), and one for ≥ one unfavorable allele(s) (heterozygote and the homozygous genotype for the minor allele). There were eight SNPs in total, and thus the scores ranged from 0 to 8 for each participant in our study.

### 2.4. i-GSEA4GWAS

Mutation data in low-grade glioma (LGG) and GBM tumors from The Cancer Genome Atlas (TCGA) were assessed using the cBioPortal for cancer genomics 63. To search for biological pathways enriched for glioma SNP associations, we performed an Improved Gene Set Enrichment Analysis for Genome-wide Association Study (i-GSEA4GWAS v1.1). SNPs up to 5 kb upstream and downstream of a given gene were mapped to that gene, and the maximum *p* value of all SNPs mapping to a gene was used to represent the gene. Gene sets used were canonical pathways, gene ontology (GO) biological process, GO molecular function, GO cellular component. As recommended, we applied an FDR cutoff of 0.10 on all reported gene sets. In the case of multiple identical pathways, that with the lower FDR value was retained.

### 2.5. Statistical Analysis

SPSS (IBM Corp. Released 2013. IBM SPSS Statistics for Windows, Version 22.0. Armonk, NY, USA) was the statistical analysis software in this research. Pearson’s chi-squared test was applied to determine significant differences between subgroups with diverse numbers of unfavorable alleles. Logistic regression analysis was conducted to evaluate both the correlation between selected SNPs and three diagnostic components of brain neoplasm, and the association between total SNP score and brain neoplasm. *p*-values below 0.05 were considered statistically significant.

## 3. Results

### 3.1. To Identify Brain Neoplasms’ SNP-Related Susceptibility Loci in Han Ancestry Populations

To determine susceptibility loci of brain neoplasm, we conducted GWAS to approach genotypes of Han populations through using the Affymetrix SNP array. Attempts to study the contribution of low-frequency variants of moderate effect have generally been carried out by sequencing candidate genes in cases of malignant and benign neoplasms; however, there are few genes associated with glioma. We sought to add more evidence to identify susceptibility SNPs modulating glioma predisposition. There have been various efforts over the past decade to investigate the contributions of small-effect variants common in the general population to many traits, including glioma-related GWAS. It is now recognized that a substantial component of glioma genetic risk is explained by combinations of common polymorphisms of modest effect [10]. Twenty-seven loci have been identified thus far from glioma GWAS [11,12].

**Figure 1:**
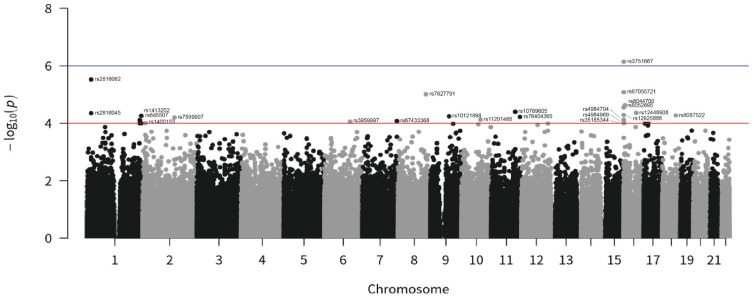
Manhattan plot for GWAS of malignant neoplasms in the Taiwanese population. Genome-wide meta-analysis *p* values (–log_10_P, *y*-axis) were plotted against their chromosomal positions (*x*-axis). All malignant (195 patients) and 195 benign (195 patients) neoplasm patients were matched for sex and age. Red and blue horizontal lines represent significance thresholds of *p* = 1 × 10^−4^.

To investigate risk factors for malignant neoplasms, we selected cases with patient ICD-10 codes C70 and C71 as the study group (malignant neoplasms); we selected cases with patient ICD-10 code D33 as the control group (benign neoplasms). After filtering, we obtained genotypes for 195 patients and 195 control subjects of Han ancestry. Collecting SNPs from GWAS resulted in a joint dataset (Table 1), and we calculated the *p* value and odds ratio (OR) under a fixed-effects model for each SNP with minor allele frequency (MAF) > 0.005. Overall and histology-specific ORs were obtained for all glioma, GBM, and non-GBM cases. According to GWAS results, associations at the established risk genes for brain neoplasms DPP6, EDARADD, ETS1, FRMD3, LMF1, LRP1B, MC4R, NDUFB9, and RBFOX1 showed a direction of effect consistent with previously reported studies. After filtering at *p* < 1 × 10^−5^ in glioma, we selected 23 SNPs for follow-up mapping to distinct loci not previously associated with brain cancer risk (*p* < 1.0 × 10^−4^, Figure 1). In the combined analysis, five SNPs showed an association with malignant-tumor risk which was genome-wide significant (Table 1): rs645507 and rs653545 (EDARADD, OR = 1.89 and 1.91; *p* = 7.71 × 10^−5^ and 1.01 × 10^−4^), rs76404385 (ETS1, OR = 0.44; *p* = 6.00 × 10^−5^), rs4984704, rs8052895, rs4984969, rs12925888, rs3751667, rs35185344, and rs67055721 (LMF1, OR = 1.86, 1.91, 1.95, 1.94, 2.17, 1.79, and 2.09; *p* = 7.57 × 10^−5^, 2.82 × 10^−5^, 9.11 × 10^−5^, 5.16 × 10^−5^, 7.24 × 10^−7^, 9.92 × 10^−5^, and 8.28 × 10^−6^), rs8087522 (MC4R, OR = −4.04; *p* = 5.35 × 10^−5^), rs8044700 (RBFOX1; OR = 2.36; *p* = 2.35 × 10^−5^), rs7599907 (LRP1B, OR = −4.00; *p* = 6.29 × 10^−5^), rs67433368 (DPP6, OR = 3.94; *p* = 8.32 × 10^−5^), rs7827791 (NDUFB9, OR = 4.42; *p* = 9.73 × 10^−6^), and rs10121898 (FRMD3, OR = −4.03; *p* = 5.70 × 10^−5^). Four signals associated with LRP1B (rs7599907), FRMD3 (rs10121898), MC4R (rs8087522), and ETS1 (rs76404385) identified benign neoplasms of the brain.

The association signals at DPP6 (rs67433368), RBFOX1 (rs8044700), LMF1 (rs3751667, rs67055721, rs4984969, rs12925888, rs8052895, rs4984704, and rs35185344), EDARADD (rs653545 and rs645507), and NDUFB9 (rs7827791) were specific for brain malignancy. DPP6 is a kind of membrane glycoprotein and shows conspicuous expression in the CNS. DPP6 enzyme activity is particularly found in humorous brain tumors [13], which suggests that DPP6 expression correlates with the formation of brain neoplasms. RBFOX1 is an RNA-binding protein (RBP) of the Fox-1 family and is involved in alternative splicing of RNAs, an especially prominent function in the brain [14]. It generates different mature messenger RNAs (mRNAs) from the same transcript, which leads to the production of more than one kind of protein from a single gene. RBFOX1 also contributes to the aggressive malignant properties of glioma [15] and regulates the blood–tumor barrier to increase permeability in glioma, eventually resulting in a lower concentration of antineoplastic medication in brain tumors [16].

Lipase maturation factor 1 (LMF1) is a specific protein that is required for lipoprotein lipase transport and maturation. LMF1 (rs3751667), which is associated with LGG, was identified in European populations [8]. Ectodysplasin A-receptor associated death domain (EDARADD) has been analyzed in human cancers in TCGA. Little is known about the mechanism of tumorigenesis in brain malignancy. NDUFB9 encodes an enzyme in the inner membrane of the mitochondria in humans and modulates mitochondrial function to affect the prognosis in cancers [17,18]. In the 2016 World Health Organization (WHO) glioma grading system, isocitrate dehydrogenase-1 (IDH-1) gene mutation was included for identifying malignancy grade in glioma. IDH gene mutation is associated with damage to mitochondrial metabolism in glioma cells [19]. Hence, NDUFB9 mutation in malignant brain tumors more so than in benign tumors suggests that mitochondrial dysfunction drives malignancy in neoplasms of the brain.

Furthermore, we also searched these genotypes in the East Asian population and found the A/G genotype at rs4984704, C/T genotype at rs8052895, A/G genotype at rs4984969, A/C/T genotype at rs12925888, C/T genotype at rs3751667, G/A/T/C deletion-insertion (delins) at rs35185344, and rs67055721 with genotypes such as G/A/T/C delins (Table 1). This alternative allele frequency in East Asia helped us to understand the mutation frequency of each SNP. Among SNPs, we observed some variation in the risk of malignant neoplasm in LD with functional variants, which is consistent with a previous polygenic model [8].

### 3.2. Relationship between Novel SNPs and the Brain Neoplasm Profile

**Figure 2:**
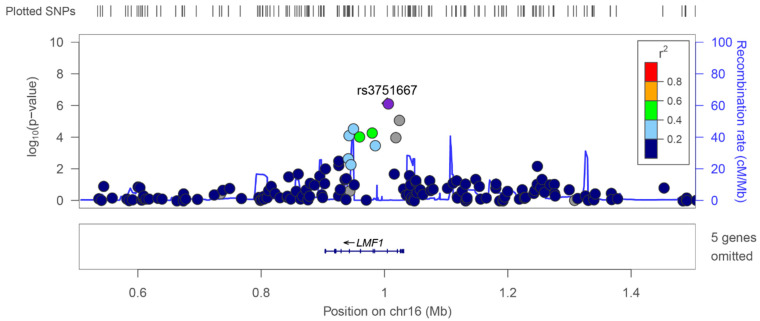
Regional association plots for brain malignancy near LMF1, showing association *p* values in discovery analysis. Purple diamonds indicate the SNPs that showed the strongest evidence of association in the malignant neoplasm datasets. Each circle in the plots indicates an SNP; the color of the circle shows the linkage disequilibrium between the SNP and the highlighted lead SNP: dark blue (r^2^ < 0.2), light blue (r^2^ > 0.2), green (r^2^ > 0.4), orange (r^2^ > 0.6), and red (r^2^ > 0.8). The r^2^ values were calculated using the genotype data from the malignant neoplasm cohort, and the recombination rate, as indicated by the blue lines in the background and the right-hand *y*-axis, was estimated from the CEU HapMap data. The bottom panels show the genes (RefSeq Genes) and their positions in each locus.

SNPs are relevant to several tumor parameters, such as classification and neoplasm grades of the brain. To further demonstrate the relevance of GWAS, we studied the correlations between the identified SNPs and malignant neoplasms. Seven SNPs at the LMF1 locus showed significant associations with malignant neoplasms (Figure 2). In addition to a previously reported locus (rs3751667), we identified genome-wide significant associations marking new risk loci for GBM at rs4984704 (*p* = 7.57 × 10^−5^), rs8052895 (*p* = 2.82 × 10^−5^), rs4984969 (*p* = 9.11 × 10^−5^), rs12925888 (*p* = 5.16 × 10^−5^), rs35185344 (*p* = 9.92 × 10^−5^), rs67055721 (*p* = 8.28 × 10^−6^), and rs8044700 (*p* = 2.35 × 10^−5^). The 16p13.3 association with malignant neoplasm that is marked by rs3751667 maps to 5_prime_UTR of LMF1, the encoded product of which belongs to an integral component of the membrane that is predicted to be active in the endoplasmic reticulum (ER) membrane. There are seven SNPs at the LMF1 locus with significant associations with malignant neoplasms (Figure 2). We used LocusZoom to view locus-specific association results at the LMF1 locus and found local linkage disequilibrium (Figure 2). A previous GWAS [8] of LGG in European populations found LMF1 to be located at locus 16p3.3, with SNP rs3751667. In addition to our novel SNP rs3751667 of LMF1, the gene is associated with the malignant brain neoplasm profile in TPMI GWAS. RBFOX1 is also located nearby (Figure 2). Notably, RBFOX1 is overexpressed in GBM, thereby playing a role in tumor viability by affecting the blood–brain barrier. Collectively, our findings provide strong evidence for specific associations for different glioma risk loci.

### 3.3. Pathway Enrichment of Glioma GWAS SNPs

**Figure 3:**
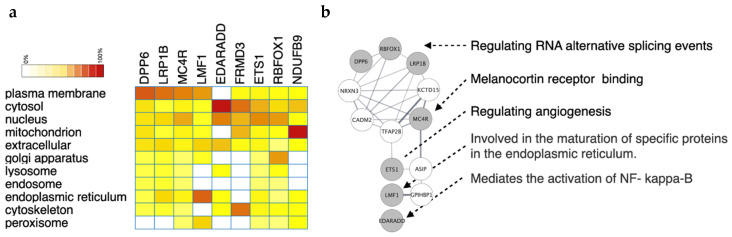
The gene–gene interaction network of crucial genes from GWAS. (**a**) Heatmap of the cellular location of GWAS-identified genes. The intensity of each cell is the frequency of appearance in GO analysis. (**b**) The protein–protein interactions of these genes are presented as a network. The circles filled with gray are GWAS-identified genes, whereas those in white were predicted by the STRING database.

Recent studies have reported that membrane proteins play an essential role in glioma formation and progression [20], which led us to consider whether these GWAS genes have some similar features in subcellular locations. To assess cellular components, we used bioinformatics to explore their subcellular locations. We examined the genes using Gene Ontology analysis and found some proteins located at the cellular membrane (Figure 3a), such as DPP6, LRP1B MC4R, and LMF1. In addition, LMF1 is predominantly localized to the ER as a membrane-bound protein. When LMF1 expresses an unfolded protein of the response target gene, it is sufficient and necessary to activate the LMF1 promoter through activating transcription factor 6 (Atf6) signaling. Most importantly, induction of LMF1 appears not restricted to lipase-expressing cells but to be a common phenomenon caused by ER stress [21]. ER stress has a synergistic contribution to EGFR inhibitor gefitinib-induced apoptosis in glioma. Gefitinib can promote one of 3 ER stress branches, namely, Atf6 [22], to target LMF1 activation. We also found that other proteins are localized to the cytosol and nucleus. Moreover, the biomolecular interaction networks constructed for GWAS-identified genes were used as input to build a protein–protein interaction network using search tool for the retrieval of interacting genes/proteins (STRING). The network was visualized in Cytoscape. In Figure 3b, the gray nodes are GWAS-identified genes; nodes in white are associated genes linked to the given genes. To gain further insight into the biological basis of associations, we performed pathway analysis on GWAS associations in brain neoplasm (Figure 3b). The protein–protein interaction (PPI) network showed DPP6, RBFOX1, and LRP1B to be related to the function of RNA alternative splicing event regulation. RBFOX1 regulates RNA alternative splicing and modulates tumorigenesis in the brain [16]. MC4R is associated with melanocortin receptor binding and EST1 with regulating angiogenesis. LMF1 is involved in the maturation of specific proteins in the ER. EDARADD is related to NF-kappa-B activation. For the PPI network, primarily signals in the malignant brain neoplasm associated with DPP6, RBFOX1, LMF1, and EDARADD to present regulation of alternative splicing of RNA by APP6 and RBFOX1, maturation of specific proteins in ER by LMF1, and activation of NF-κB through EDARADD. On the other hand, MC4R and ETS1 in benign brain tumor are primarily associated with melanocortin receptor binding and regulating angiogenesis (Figure 3b).

### 3.4. Survival Analysis of Malignant Neoplasms in Taiwan

**Figure 4:**
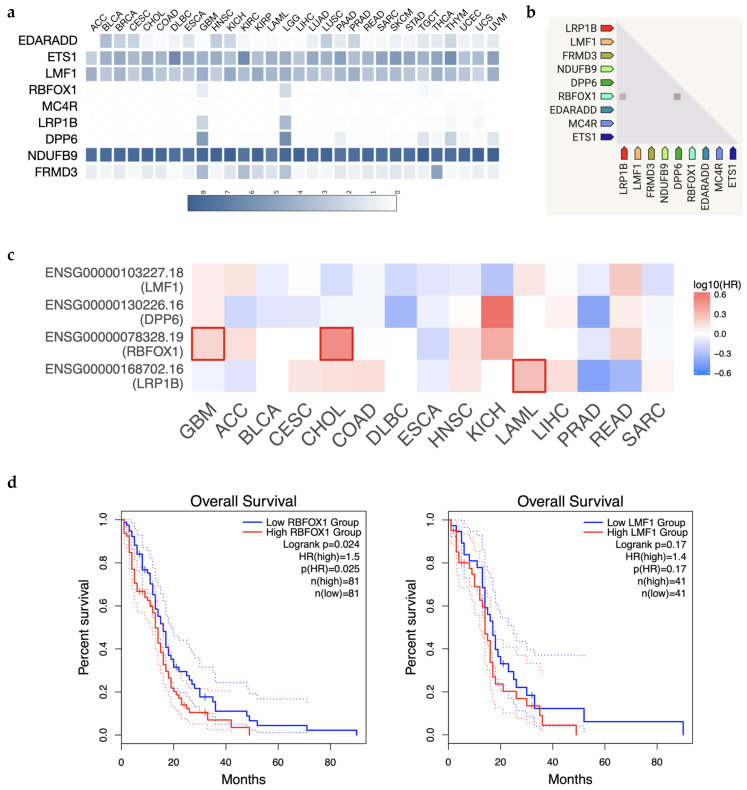
High RBFOX1 and LMF1 expression is associated with worse prognosis in glioblastoma. (**a**) Expression of GWAS-identified genes in various cancer types. The expression intensity is presented as log2(TPM + 1). (**b**) The relationship among GWAS-identified gene expression correlates across a large number of tumor experiments. (**c**) The survival map of LMF1, LRP1B, RBFOX1, and DPP6. The color of each cell is presented by the hazard ratio. The significant hazard ratio is labeled by a bold box. (**d**) Kaplan–Meier curves show the impact of RBFOX1 and LMF1 expression on the survival of all GBM patients from TCGA.

According to the epidemiology of brain neoplasms in the Taiwan Cancer Registry, Annual Report 2018, the most common pathology of malignant neoplasms was glioblastoma The data showed that glioma is the cumulative common histopathology of brain cancer. Unfortunately, there are few data on survival of malignant brain tumors in the Han population. In the TPMI, most cases appear to be glioma because the most common prevalence of brain malignancy in Taiwan is glioma. To investigate the risk due to the GWAS-identified genes, we collected patient data from TCGA. First, we found DPP6, LRP1B, and RBFOX1 to be solely expressed in brain cancer (GBM and LGG) but not in all cancer mutations (ACC, BLCA, BRCA, CESC, CHOL, DLBC, ESCA, HNSC, KICH, KIRP, LAML, LIHC, LUAD, LUSC, PAAD, PRAD, READ, SARC, SKCM, STAD, TGCT, THCA, THYM, UCEC, UCS, and UVM), suggesting a brain-specific role in tumorigenesis (Figure 4a). Of all cancer types in TCGA, these three genes are highly associated and share similar expression patterns (Figure 4b). We further confirmed the clinical impact of these genes by examining survival rate data. Overall, survival rate analysis showed that the level of RBFOX1 significantly impacts GBM survival the most, but that LMF1 and DPP6 have a moderate effect on GBM. We further validated their effect on survival in detail, whereby a high RBFOX1 level strongly reduced the survival rate in 162 GBM patients (*p* = 0.024; HR = 1.5, Figure 4d left). Similarly, LMF expression reduced survival in GBM patients (*p* = 0.17; HR = 1.4, Figure 4d right). Taken together, among the four novel brain malignancy genes from GWAS (LMF1, DPP6, RBFOX1, and LRP1B), RBFOX1 may be the crucial effector in GBM progression.

## 4. Discussion

### 4.1. Possible Mechanisms That Link These SNPs with Malignant Brain Tumors

Researchers have performed several GWASs of glioma and found glioma susceptibility loci at 12q23.33, 10q25.2, 11q23.2, 12q21.2, and 15q24.2, for a total of 12 risk loci. However, such studies focusing on the Han Chinese population are still scarce. Here, we provided updated information on brain malignancy, identifying a crucial effector among susceptible genes. Considering the multifactorial etiology of brain cancer, identification of genetic factors that are involved in the disease trajectory is popular. Nevertheless, results to date have varied owing to different sample groups or analytic methods, revealing a large number of unknowns in this field. RBFOX1 can regulate alternative splicing in the brain [14]. RBFOX1 is an RBP, which promotes the maturation of mRNAs. Mutated RBFOX1 contributes to the aggressive malignant properties of glioma [15]. RBFOX1 further regulates the blood–tumor barrier to increase permeability in the glioma and may serve as a target to provide a higher concentration of antineoplastic medication in brain cancer [16]. An increasing number of RBPs have a crucial role in tumorigenesis. A noteworthy RBP in brain malignancy is Musashi-1 (MSI1), which promotes radioresistant GBM escape from apoptosis and enhances homologous recombination DNA repair [23]. MSI1 causes tumor migration by binding with ICAM1 RNA, leading to poor prognosis [24]. MSI1 can be inhibited by microRNAs to reduce brain carcinogenesis [25]. RBFOX1 could not be confirmed based on previous studies to have positive or negative correlations with gliomagenesis. However, RBFOX1 (rs8044700) near LMF1 in our TPMI study has an alternative splicing function to modify brain neoplasms and tend to be associated with malignancy.

A GWAS in glioma [8] in European populations found LMF1 (rs3751667) at 16p3.3 to be involved in LGG. This result is similar to our TPMI study for malignant neoplasms of the brain in the Han population. LMF1 is activated in the ER and regulates ER stress. Malignancy is produced by ER stress through an ongoing unfolded protein response [26]. GRP78, an ER protein, further regulates glioma cell proliferation and apoptosis, and ER stress might cause these proteins to unfold to overcome cell death [27]. Protein interactions also showed that LMF1 is involved in the maturation of specific proteins in the ER. Thus, LMF1 in the Han Chinese population is predominantly related to malignant neoplasms of the brain and might predispose an individual toward brain cancer by regulating ER stress.

Other SNP profiles revealed different molecules, including DDP6, NDUFB9, EDARADD, EST1, MC4R, LRP1B, and FRMD3, which perform different roles in brain neoplasms or malignancies. A previous study [28] involving analysis of TCGA data reported that DPP6 induces tumorigenesis in GBM and correlates with prognosis in clear cell renal cell carcinoma [29]. DDP6 mutation is more predominant in malignant neoplasms of the brain in the Han population. NDUFB9 modulates mitochondrial function to affect the prognosis of cutaneous melanoma [17] and uveal melanoma [18]. Mutated or wild-type IDH-1 might determine glioma grade, and mitochondrial dysfunction is associated with IDH gene mutation in glioma [19]. Thus, NDUFB9 might induce those brain neoplasms to become malignant or benign via regulation of mitochondrial metabolism.

In several cohort studies [30,31], high EDARADD expression was associated with poor diagnosis and a high recurrence rate in ovarian cancer, tongue squamous cell carcinoma, prostate cancer, and colorectal malignancy [31,32,33]. EDARADD DNA methylation is overall associated with high tumor recurrence and poor prognosis. DNA methylation in GBM presents a significant relationship with disease control and overall survival, and methylation of the repair enzyme O6-methylguanine-DNA methyltransferase (MGMT) gene enhances the response to concurrent chemoradiotherapy with temozolomide (TMZ). Thus, methylated MGMT in GBM may lead to better efficacy of treatment and improved prognosis [34]. According to the results of previous research, DNA methylation of EDARADD in brain cancer might predict therapeutic response.

As a transcriptional regulator, EST1 not only promotes glioma cell transition to the mesenchymal type but is also activated by c-Met to remodel vascular endothelial cells [35,36], driving aberrant vascularization and chemoresistance in glioblastoma [37]. MC4R drives a cell toward a relatively stable equilibrium between energy sources, including glucose and lipids. MC4R inhibition in GBM overcomes cell proliferation and promotes apoptosis via downregulation of ERK1/2 and Akt phosphorylation. At the same time, MDC4R inhibition induces a synergistic effect in combination therapy with temozolomide in GBM [38].

LRP1B is a member of the low-density lipoprotein receptor-related protein family and is associated with metabolic stress in cancer. LRP1B deletions with different molecular alterations, including FAM72, affect the prognosis of GBM patients in TCGA [39,40]. LRP1B enhances expression of CD133, a cancer stem cell marker of brain neoplasms, to affect serum-starved medulloblastoma [41]. However, there is no association between GBM patient outcomes and LRP1B mRNA levels [40]. From our primary analysis of the TPMI dataset for the Han population, LRP1B mutation showed more significant expression in benign neoplasms than in malignant neoplasms of the brain, suggesting that LRP1B acts as a tumor-suppressor gene (TSG) in benign brain tumors.

FRMD3, which is alternatively called band 4.1-like protein 4° [42], is expressed at low levels in the brain and a 4.1B type of the protein 4.1 family. FRMD3 is considered a putative TSG [43]. Biochemically unique FERM (F for 4.1 protein, E for ezrin, R for radixin, and M for moesin) proteins have been ascribed to some new physiological functions in the muscle, ovary, thyroid, and brain. Mutations in the FERM domain of Pyk2 inhibit phosphorylation. The protein-to-protein interaction between MAP4K4 and the Pyk2 FERM domain regulates cell motility in glioma cells [44]. Despite limited specific clinical research between FRMD3 and brain neoplasms, FERMD might be relevant as a TSG in benign neoplasms of the brain, as indicated by our TPMI results in the Han population.

Both LRP1B and FRMD3 serve as TSGs in brain tumors, and some research has shown that LRP1B deletion affects the prognosis of malignant brain neoplasms, including GBM and medulloblastoma, in the U.S. population according to TCGA data [39,40,41]. However, little is known about FRMD3 in brain cancer. A previous study of FRMD3 mentioned that high levels of FRMD3 in rectal cancer lead to a poor disease survival rate [45]. FRMD3 was also found to be a candidate TSG in lung cancer and acute myeloid leukemia [43,46]. Although there is little research on FRMD3 in neoplasms of the brain, FRMD6 inhibits activation of tyrosine kinase receptors to overcome tumor growth and disease progression in GBM [47].

### 4.2. Limitations of Our Study

Our current study has several limitations. First, our participants were recruited from a single medical institution in Taiwan, ROC. All were Taiwanese adults aged above 40 years old. Due to the limited ethnicity and range of age sampling, the generalizability of our results may be unconvincing. Further study is warranted to extend the applicability of GWAS in glioma and validate the replication of existing studies.

## 5. Conclusions

Taken together, previous findings, and ours, suggest various SNPs and genes that are relevant for brain cancer genomics. Despite the absence of a common result, we discussed potential mechanisms that link these genes with brain cancer in the hope that future studies will obtain the full picture. In conclusion, our study found brain neoplasm-related SNPs that have not yet been reported. Among them, rs8044700 participates in RNA modification, and rs3751667 induces cellular ER stress. Although not directly associated with brain tumors, a shared underlying mechanism may be inferred. Therefore, both mechanisms for brain carcinogenesis are important for the Han population. One is regulating RNA splicing events through RBFOX1 and the other is ER stress-related LMF1. These findings should provide the research motivation for drug discovery in the future. Finally, those patients with malignant neoplasms of the brain might consider arranging examinations for those biomarkers or SNPs. Then, to determine the precision medicines, we ought to focus on the maturation of proteins in the ER or RNA modifications for them. Other SNPs mostly located in intergenic regions require further study to assess their functions.

## Figures and Tables

**Table 1:** Summary of the characteristics of SNPs in malignant neoplasms.

SNP	Symbol	Nearest Gene	Cytogenetic Band	Loci (GRCh38.p13)	All Cases		East Asia
					*p*	OR	Alternative Allele
rs2816045	-	PAX7	1p36.13	1:18566505-18566505	4.44 × 10^−5^	1.86	G = 0.3800
rs2816062	-	PAX7	1p36.13	1:18577408-18577408	2.99 × 10^−6^	2.03	A = 0.3720
rs645507	EDARADD	-	1q42.3	1:236409960-236409960	7.71 × 10^−5^	1.89	A = 0.3167
rs653545	EDARADD	-	1q42.3	1:236422530-236422530	1.01 × 10^−4^	1.91	C = 0.3000
rs1413202	-	PLD5	1q43	1:242708730-242708730	5.49 × 10^−5^	1.83	C = 0.4033
rs34992957	-	PLD5	1q43	1:242712199-242712199	1.01 × 10^−4^	1.83	C = 0.3400
rs11201468	-	ENST00000656796	10q23.1	10:85206976-85206976	7.47 × 10^−5^	0.31	T = 0.0500
rs10789605	-	ENSG00000261098	10q23.1	11:107313859-107313859	3.97 × 10^−5^	0.51	G = 0.2867
rs76404385	ETS1	-	11q24.3	11:128463160-128463160	6.00 × 10^−5^	0.44	T = 0.152
rs4984704	LMF1	-	16p13.3	16:890713-890713	7.57 × 10^−5^	1.86	G = 0.354
rs8052895	LMF1	-	16p13.3	16:897817-897817	2.82 × 10^−5^	1.91	T = 0.382
rs4984969	LMF1	-	16p13.3	16:907885-907885	9.11 × 10^−5^	1.95	G = 0.293
rs12925888	LMF1	-	16p13.3	16:927944-927944	5.16 × 10^−5^	1.94	G = 0.34
rs3751667	LMF1	-	16p13.3	16:954554-954554	7.24 × 10^−7^	2.17	T = 0.412
rs35185344	LMF1	-	16p13.3	16:966608-966612	9.92 × 10^−5^	1.79	ATATA = 0.44
rs67055721	LMF1	-	16p13.3	16:972244-972249	8.28 × 10^−6^	2.09	C = 0.34
rs8044700	RBFOX1	-	16p13.3	16:6093700-6093700	2.35 × 10^−5^	2.36	A = 0.84
rs12448908	-	FTO	16q12.2	16:54205958-54205958	4.64 × 10^−5^	0.53	C = 0.69
rs8087522	MC4R	-	18q21.32	18:60373245-60373245	5.35 × 10^−5^	0.37	A = 0.123
rs1400153	**-**	ENSG00000285876	2p24.3	2:13029448-13029448	9.79 × 10^−5^	0.57	G = 0.472
rs7599907	LRP1B	-	2q22.2	2:141433506-141433506	6.29 × 10^−5^	0.52	C = 0.70
rs3959997	-	FRK	6q22.1	6:115838393-115838393	8.82 × 10^−5^	0.48	T = 0.19
rs67433368	DPP6	-	7q36.2	7:154353490-154353490	8.32 × 10^−5^	3.13	A = 0.01
rs7827791	NDUFB9	-	8q24.13	8:124549270-124549270	9.73 × 10^−6^	1.91	G = 0.52
rs10121898	FRMD3	-	9q21.32	9:83324595-83324595	5.70 × 10^−5^	0.46	A = 0.07

## Data Availability

The datasets used and/or analyzed during the current study are available from the corresponding author on request. This will in most cases also require an ethical permit.
